# Nanoformulations of Ursolic Acid: A Modern Natural Anticancer Molecule

**DOI:** 10.3389/fphar.2021.706121

**Published:** 2021-07-05

**Authors:** Longyun Wang, Qianqian Yin, Cun Liu, Ying Tang, Changgang Sun, Jing Zhuang

**Affiliations:** ^1^ College of Traditional Chinese Medicine, Shandong University of Traditional Chinese Medicine, Jinan, China; ^2^ College of First Clinical Medicine, Shandong University of Traditional Chinese Medicine, Jinan, China; ^3^ Department of Hematology, Affiliated Hospital of Weifang Medical University, Weifang, China; ^4^ Department of Oncology, Weifang Traditional Chinese Hospital, Weifang, China; ^5^ Qingdao Academy of Chinese Medical Sciences, Shandong University of Traditional Chinese Medicine, Qingdao, China

**Keywords:** ursolic acid, natural molecule, cancer, pharmacokinetics, nanoformulations

## Abstract

**Background:** Ursolic acid (UA) is a natural pentacyclic triterpene derived from fruit, herb, and other plants. UA can act on molecular targets of various signaling pathways, inhibit the growth of cancer cells, promote cycle stagnation, and induce apoptosis, thereby exerting anticancer activity. However, its poor water-solubility, low intestinal mucosal absorption, and low bioavailability restrict its clinical application. In order to overcome these deficiencies, nanotechnology, has been applied to the pharmacological study of UA.

**Objective:** In this review, we focused on the absorption, distribution, and elimination pharmacokinetics of UA in vivo, as well as on the research progress in various UA nanoformulations, in the hope of providing reference information for the research on the anticancer activity of UA.

**Methods:** Relevant research articles on Pubmed and Web of Science in recent years were searched selectively by using the keywords and subheadings, and were summarized systematically.

**Key finding:** The improvement of the antitumor ability of the UA nanoformulations is mainly due to the improvement of the bioavailability and the enhancement of the targeting ability of the UA molecules. UA nanoformulations can even be combined with computational imaging technology for monitoring or diagnosis.

**Conclusion:** Currently, a variety of UA nanoformulations, such as micelles, liposomes, and nanoparticles, which can increase the solubility and bioactivity of UA, while promoting the accumulation of UA in tumor tissues, have been prepared. Although the research of UA in the nanofield has made great progress, there is still a long way to go before the clinical application of UA nanoformulations.

## Introduction

### A Close Connection Between UA and Cancer

**FIGURE 1:**
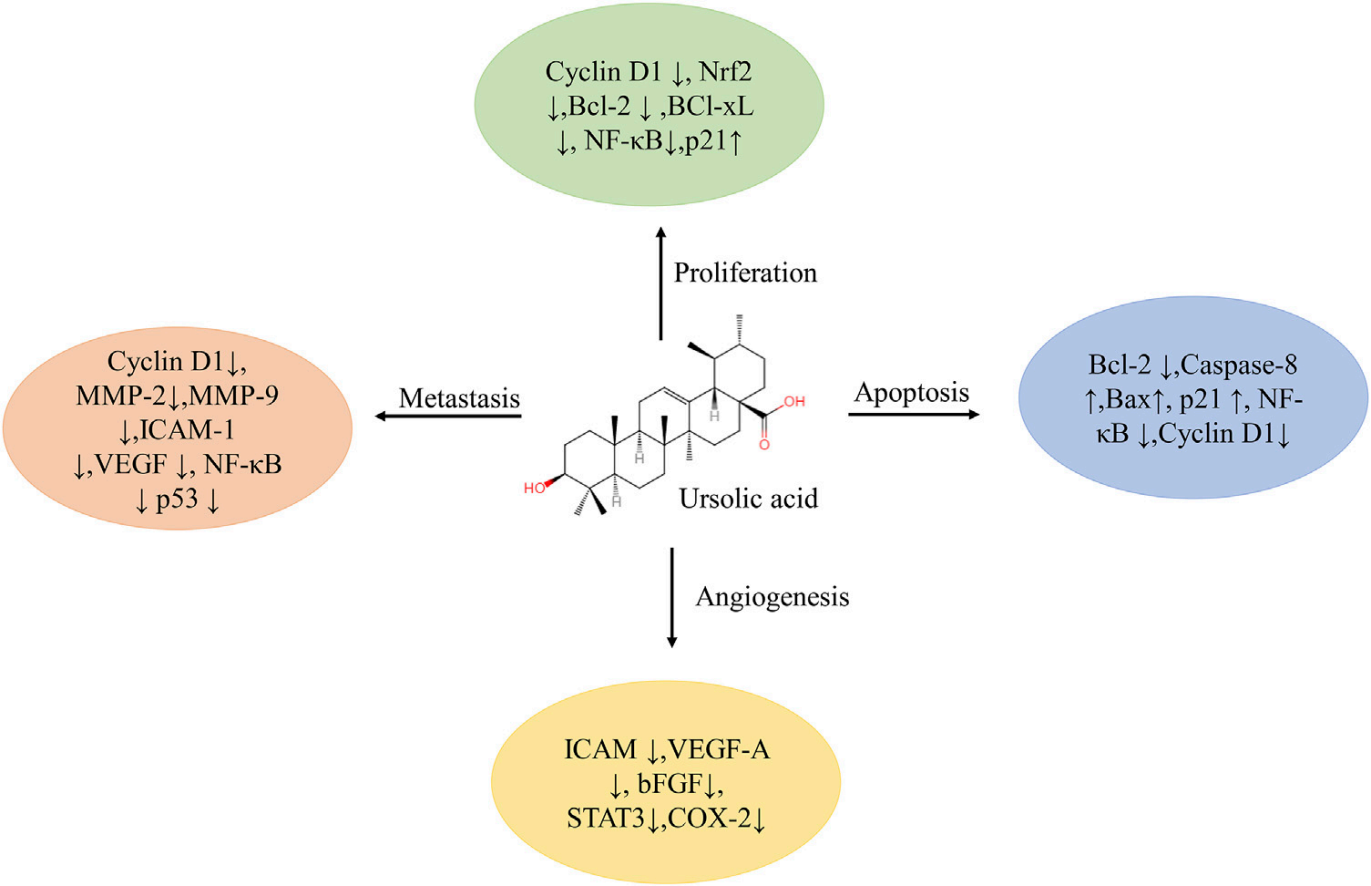
Multiple molecular targets modulated by ursolic acid. Abbreviations: Bcl-2, B-cell lymphoma-2; Bcl-xL, B-cell Lymphoma/Leukemia-xL; bFGF, basic fibroblast growth factor; COX, cyclooxygenase; ICAM, intercellular adhesion molecule; MMP, matrix metalloprotease; NF-κB, nuclear factor kappa-B; Nrf, nuclear factor E2-related factor; STAT, signal transducers and activators of transcription; VEGF, vascular endothelial growth factor.

Cancer is a complex disease caused by the abnormal proliferation of cells under the action of multiple factors (Yu et al., 2018). Over the years, natural products, with significant anticancer potential and the ability to act as bioenhancers to increase the activity of existing anticancer drugs, have been an unrivaled medicine source (Choudhari et al., 2019; Dwivedi et al., 2019). Data show that more than 70% of FDA-approved cancer drugs come from natural products or natural product derivatives (Katz and Baltz, 2016; Cargnin and Gnoatto, 2017). Natural compounds can inhibit the formation and development of cancer through specific interactions with a variety of cell signaling pathways, thereby confirming the intriguing anticancer effectiveness of natural products (Calcabrini et al., 2017). Studies have shown that the natural molecule ursolic acid (UA) can regulate the proliferation, metastasis, angiogenesis, and apoptosis of tumor cells by acting on a variety of cytokines (Figure 1), and has a significant therapeutic effect on breast, lung, colorectal, liver, and prostate cancer as well as on other tumors (Mu et al., 2018; Zhang et al., 2020a; Gou et al., 2020b; Chen et al., 2020; Shao et al., 2020). The detailed roles of UA in the treatment of cancer are shown in Table 1.

**TABLE 1:** The detailed role of UA and cancer.

Types of cancer	Cell line used	Study type	Therapeutic effect	Mechanism of action	References
Breast Cancer	MCF-7	*In vitro*	Inhibit cell growth	Down-regulate the phosphorylation of PLK1	Guo et al. (2020a)
				Inhibit RAF/ERK pathway and IKK/NF-κB pathway	
	MCF-7	*In vitro*	Inhibit invasiveness and migration	Inhibit PMA-induced MMP-9 expression	Chung et al. (2017)
			Induce apoptosis	Down-regulate the phosphorylation of p-ERK and p-p38	
				activate caspase-8, caspase-7 and poly ADP-ribose polymerase; up-regulate the expression of Bax and down-regulate the expression of Bcl-2	
	MDA-MB-231	*In vitro*	Inhibit cell proliferation	Down-regulate Nrf2 via Keap1/Nrf2 pathway and EGFR/Nrf2 pathway	Zhang et al. (2020a)
	MCF-7, MDA-MB-231	*In vitro*	Control cell proliferation; induce apoptosis	Inhibit the activity of CDK6	Yousuf et al. (2020)
	MCF-7	*In vitro*	Inhibit cell survival	–	Lam et al. (2019), Ngo et al. (2019)
	184-B5,184-B5/HER	*In vitro*	Induce apoptosis	Down-regulate the expression of Bcl-2; up-regulate the expression of Bax	Telang, (2018)
	T47D, MCF-7, MDA-MB-231	*In vitro*	Inhibit cell proliferation Induce autophagy and apoptosis	Down-regulate the expressions of Bcl-2, cyclin-D1 and NF-κB	Luo et al. (2017)
				Up-REGULATE GSK through decreasing PI3K/AKT pathway	
	SUM149PT, HCC 1937, MDA-MB-231	*In vitro*	Induce cell cycle arrest; induce apoptosis	–	Gu et al. (2017); Li W. et al. (2018)
	MDA-MB-231	*In vitro*	Induce cell cycle arrest; induce apoptosis	Down-regulate PCNA, CDK4, and Cyclin-D1; up-regulate p21^Waf1/Cip1^	Wen et al. (2017)
				Induce the activation of caspase-9 and caspase-3 by mitochondrial death pathway	
	HCC1806	*In vitro*	Induce cell cycle arrest; induce apoptosis	Down-regulate the activation of STAT3	Li W. et al. (2017)
				Up-regulate the expressions of p21 and p27	
	MCF-7, MDA-MB-231, SK-BR-3	*In vitro*	Inhibit cell proliferation	Up-regulate the levels of p21, superoxide and protein carbonylation	Lewinska et al. (2017b)
				Down-regulate the levels of NOP2, p120 and WDR12	
	MCF-7, MDA-MB-231, SK-BR-3	*In vitro*	Induce autophagy and apoptosis	Up-regulate the levels of p21	Lewinska et al. (2017a)
				Down-regulate the levels of HK2, PKM2, ATP and lactate via AKT signaling pathway	
	Mice xenografted with MMTV-Wnt-1 mammary tumor cells	*In vivo*	Reduce tumor volume; induce apoptosis and arrest cell cycle	Modulate Akt/mTOR signaling pathways	(De Angel et al., 2010; Wang M. et al. (2020)
Lung Cancer	A549, H460	*In vitro*	Induce cell cycle arrest	Up-regulate the levels of CHOP, Bax and caspase-8 through ER stress pathway	Gou et al. (2020a)
			Induce apoptosis		
	A549	*In vitro*	Inhibit autophagy	INHIBIT the mTOR signaling pathway	Wang M. et al. (2020)
	H549	*In vitro*	Induce apoptosis	Activate AKT/mTOR pathway	Castrejón-Jiménez et al. (2019)
	H292	*In vitro*	Inhibit cell survival	Up-regulate the expression levels of AIF and Endo G through a mitochondria-dependent pathway	Chen et al. (2019)
			Induce apoptosis		
	A549	*In vitro*	Induce cell cycle arrest	Block the NF-κB signaling pathway	Jiang et al. (2018)
			Induce apoptosis		
	H1975	*In vitro*	Inhibit invasiveness and metastasis	Decrease the level of E-cadherin and elevate the level of N-cadherin through TGF-β1 signaling pathway	Ruan et al. (2019)
				Decrease the levels of MMP-2 and MMP -9	
	H460	*In vitro*	Inhibit cell survival	Activate the levels of caspase-8 and caspase-7 and decrease the level of Bcl-2	Mendes et al. (2016)
			Induce apoptosis	Increase the levels of Beclin-1 and LC3A/B-II and decrease the level of mTOR and p62	
	H1299, A549, H1650, H358, H1975	*In vitro*	Inhibit cell growth	Induce phosphorylation of SAPK/JNK and suppress the protein expression of DNMT1 and EZH2	Wu et al. (2015)
			Induced apoptosis		
Colorecta-l Cancer	RKO	*In vitro*	Inhibit cell proliferation; induce apoptosis	Increase the activities of caspase-3, caspase-8, and caspase-9	Zheng et al. (2020)
	HCT116, HCT-8	*In vitro*	Inhibit cell proliferation and angiogenesis	Regulate the TGF-β1/ZEB1/miR-200c signaling pathway	Zhang L. et al. (2019)
	SW620, HCT116	*In vitro*	Inhibit cell proliferation and metastasis	Inhibit the biomakers of EMT including E-cadherin, Vimentin, Integrin, Twist, and Zeb1	Wang et al. (2019)
	HCT116, HT29	*In vitro*	Induce apoptosis	Up-regulate the expression levels of MicroRNA-4500	Kim et al. (2018)
				Inhibit the phosphorylation of JAK2/STAT3	
	SW480, SW620, LoVo, RKO, SW620 xenograft mouse model	*In vitro, in vivo*	Inhibit cell proliferation Induced apoptosis	Down-regulate Bcl-xL, Bcl-2 and surviving	Shan et al. (2016)
				Activate caspase-3, 8, 9; inhibit the expression levels of KRAS and BRAF, MEK1/2, ERK1/2, p-38, JNK, AKT, IKKα, IκBα, and p65 phosphorylation of the MAPK, PI3K/AKT, and NF-κB signaling pathways	
	CaCo-2	*In vitro*	Induced apoptosis	Activate the expression of Caspase 3	Omoyeni et al. (2015)
	HT-29, CRC mouse xenograft model	*In vitro, in vivo*	Inhibit angiogenesis	Inhibit the expressions of VEGF-A and bFGF	Lin et al. (2013a)
				Suppress the activation of SHH, STAT3, akt and p70S6K pathways	
	HT-29, CRC mouse xenograft model	*In vitro, in vivo*	Inhibit cell proliferation	Modulate the expressions of Cyclin D1, CDK4 and p21	Lin et al. (2013b)
			Induced apoptosis	alter the ratio of Bax/Bcl-2; activate of several CRC-related signal transduction cascades including STAT3, ERK, JNK and p38	
			Inhibit tumor growth		
	HCT15, Nude mice xenografted with HCT15 cells	*In vitro, in vivo*	Induce cell death	Activate the JNK pathway	Xavier et al. (2013)
			Modulate autophagy		
	Orthotopic nude mouse model	*In vivo*	Inhibit cell growth and metastasis	Inhibit the activation of constitutive NF-κB	Prasad et al. (2012)
				Down-regulate Bcl-xL, Bcl-2, cFLIP, surviving, cyclin D1, MMP-9, VEGF, ICAM-1, EGFR, p53 and p21	
				Down-regulate Ki-67 and CD31 accompanied by suppression of NF-κB, STAT3, and *β*-catenin	
	HT-29	*In vitro*	Induce apoptosis	Activate the P2Y2/Src/p38/COX-2 pathway	Limami et al. (2012)
Liver Cancer	HepG2	*In vitro*	Inhibit cell survival	–	(Lam et al., 2019; Ngo et al., 2019)
	HepG2, 7721, HuH7	*In vitro*	Inhibit cell growth	Down-regulate the expression of downstream target genes of STAT3, such as Bcl-2, Bcl-xl and surviving	Liu et al. (2017)
	HepG2	*In vitro*	Inhibit cell growth	Inhibit growth through AMPKα-mediated reduction of DNA methyltransferase 1	Yie et al. (2015)
	HepG2	*In vitro*	Induce apoptosis	activate the phosphorylation of AMPK and GSK3β	Son et al. (2013)
	Hep3B, HuH7, HA22T		Inhibit invasiveness and metastasis	Decrease the levels of VEGF, IL-8, ROS and NO	Lin et al. (2011)
				Retain the level of glutathione	
	SMMC-7721		Induce apoptosis	Activate p53-dependent pathway	Yu et al. (2010)
Prostate Cancer	LNCaP	*In vitro*	Induce apoptosis	Activate caspase-3/9 via mediation of ROCK1/PTEN-cofilin-1/cytochrome c protein expression	Mu et al. (2018)
	LNCaP, PC-3 xenograft mouse model with LNCaP/PC-3 cells	*In vitro, In vivo*	Induce apoptosis	Decrease the levels of Bcl-2, Bcl-xl, and surviving	Meng et al. (2015)
				Activate caspase-3; activate the PI3K/Akt/mTOR pathway	
Renal Cancer	A498	*In vitro*	Inhibit invasiveness	Up-regulate the expression levels of NLRP3, caspase-1 and IL-1β	Chen et al. (2020)
	786-0	*In vitro*	Induce cell cycle arrest	Inhibit the activation of STAT3 and the expressions of p21 and p27	Li W. et al. (2017)
			Induce apoptosis		

*Abbreviations* ADP, adenosine diphosphate; AIF, apoptosis-inducing factor; AKT, protein kinase B; AMPK, adenosine 5‘-monophosphate (AMP)-activated protein kinase; ATP, adenosine triphosphate; Bax, Bcl-2-associated X protein; Bcl-2, B-cell lymphoma-2; Bcl-xL, B-cell Lymphoma/Leukemia-xL; bFGF, basic fibroblast growth factor; BRAF, v-raf murine sarcoma viral oncogene homolog B1; CDK, cyclin-dependent kinases; cFLIP, Fas-associated death domain-like interleukin-1β-converting enzyme (FLICE)-like inhibitory protein; CHOP, endoplasmic reticulum stress pathway marker protein; COX, cyclooxygenase; DNMT1, DNA (cytosine-5)-methyltransferase 1; EGFR, epithelial growth factor receptor; EMT, epithelial-mesenchymal transition; ER, endoplasmic reticulum; ERK, extracellular regulated protein kinases; EZH2, enhancer of zeste 2 polycomb repressive complex 2 subunit; GSK, glycogen synthase kinase; HK, hexokinase; ICAM, intercellular adhesion molecule; IKK, inhibitor of nuclear factor kappa-B kinase; IL, interleutin; JAK, Janus kinase; JNK, jun N-terminal kinase; Keap1, Keal-like ECH-associated protein 1; KRAS, V-Ki-ras2 Kirsten ratsarcoma viral oncogene homolog; MAPK, mitogen-activated protein kinase; MEK1/2, MAP kinase kinase 1/2; MMP, matrix metalloproteinase; mTOR, mammalian target of rapamycin; NF-κB, nuclear factor kappa-B; NLRP3, NLR family pyrin domain-containing 3; NO, nitric oxide; Nrf, nuclear factor E2-related factor; PCNA, proliferating cell nuclear antigen; p-ERK, phosphorylated extracellular regulated protein kinases; PI3K, phosphoinositide 3-kinase; PKM2, pyruvate kinase; PLK, polo-like kinase; PMA, phorbol myristate acetate; p-p38, phosphorylated p38 mitogen-activated protein kinase; PTEN, phosphatase and tensin homolog; P2Y2, specific purinergic receptors belonging to the P2Y families; RAF, RAF serine/threonine kinase protein; ROCK, Rho-associated protein kinase; ROS, reactive oxygen species; SAPK, stress-activated protein kinase; SHH, sonic hedgehog; Src, Src protein tyrosine kinase; STAT, signal transducers and activators of transcription; TGF-β1, Transforming growth factor-β1; UA, ursolic acid; VEGF, vascular endothelial growth factor; ZEB1, zinc finger E-box-binding homeobox.

### The Source and Characteristics of UA Molecule

Ursolic acid (3β-hydroxy-urs-12-en-28-oic-acid, PubChem CID:64945, CAS:77-52-1, Figure 2), with the molecular formula C_30_H_48_O_3_ and a molecular weight of 456.7 g/mol, is a natural arbutin pentacyclic triterpenoid, which is widely found in fruits, medicinal materials, and other plants (Son and Lee, 2020). Pentacyclic triterpenes (PTs) are natural secondary metabolites of plants and many other organisms. First discovered in the 1920s from apple epidermis wax, UA has been isolated from many other plant organs in recent years. Examples include pears, olives, plums, cranberries, *Fructus Chaenomelis Lagenariae, Fructus Mume*, *Fructus Gardeniae*, *Fructus Ligustri Lucidi*, and *Hedyotis diffusa Willd*, among others (Ikeda et al., 2008; Sun Q. et al., 2020). This compound has a wide range of biological and pharmacological properties, in addition to attractive functional cytotoxic properties, including anti-inflammatory, antioxidant, anti-allergy, antiviral, antibacterial, liver protection, sedative, anti-ulcer, lipid-lowering, and anti-diabetic effects (Wan et al., 2019). It is worth noting that although UA is a promising bioactive molecule, there are still many obstacles that need to be overcome before it can truly reach its potential.

**FIGURE 2:**
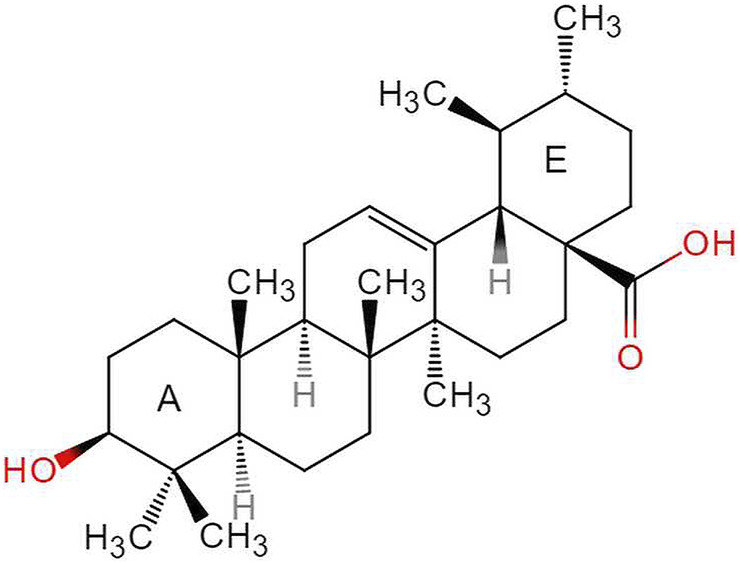
The structure of ursolic acid.

The pentacyclic terpene UA structure (C-30 isoprenoid, alcoholic NaOH, and glacial acetic acid) has good solubility; however, its solubility in water is low, leading to poor oral drug absorption in the body, short half-life, and low bioavailability. These disadvantages limit its wide application in the pharmaceutical field, and it has been classified as class IV in the biological drug classification system (BCS) (Yu et al., 2020). UA has been studied extensively in efforts to overcome its pharmacological limitations and enhance its therapeutic effects; some research methods have included structural modification or nanomaterial preparation. Kalani et al. verified the significant time-dose dependent anti-cancer activity of UA derivatives through quantitative structure-activity relationship (Kalani et al., 2012; Kalani et al., 2014). Khwaza et al. (2020) summarized the anticancer activity of UA derivatives after structural modification, showing that the molecular structure modification of UA mainly involves three sites: the C28 carboxyl group, the C3 hydroxyl group, and the C12–C13 double bond. However, it is noteworthy that not all new UA derivatives exhibit enhanced anticancer activity. On the contrary, some exhibit decreased anticancer activity, which may be due to the different modification sites or pathways of UA (Manayi et al., 2018). UA nanoformulations change the pharmacokinetic properties of UA by reducing the particle size, changing the surface performance, and improving stability, thus achieving the effect of improving its anticancer activity and reducing adverse reactions.

### The Basic Strategy of Nanoformulations Applied to Tumor Tissues

**FIGURE 3:**
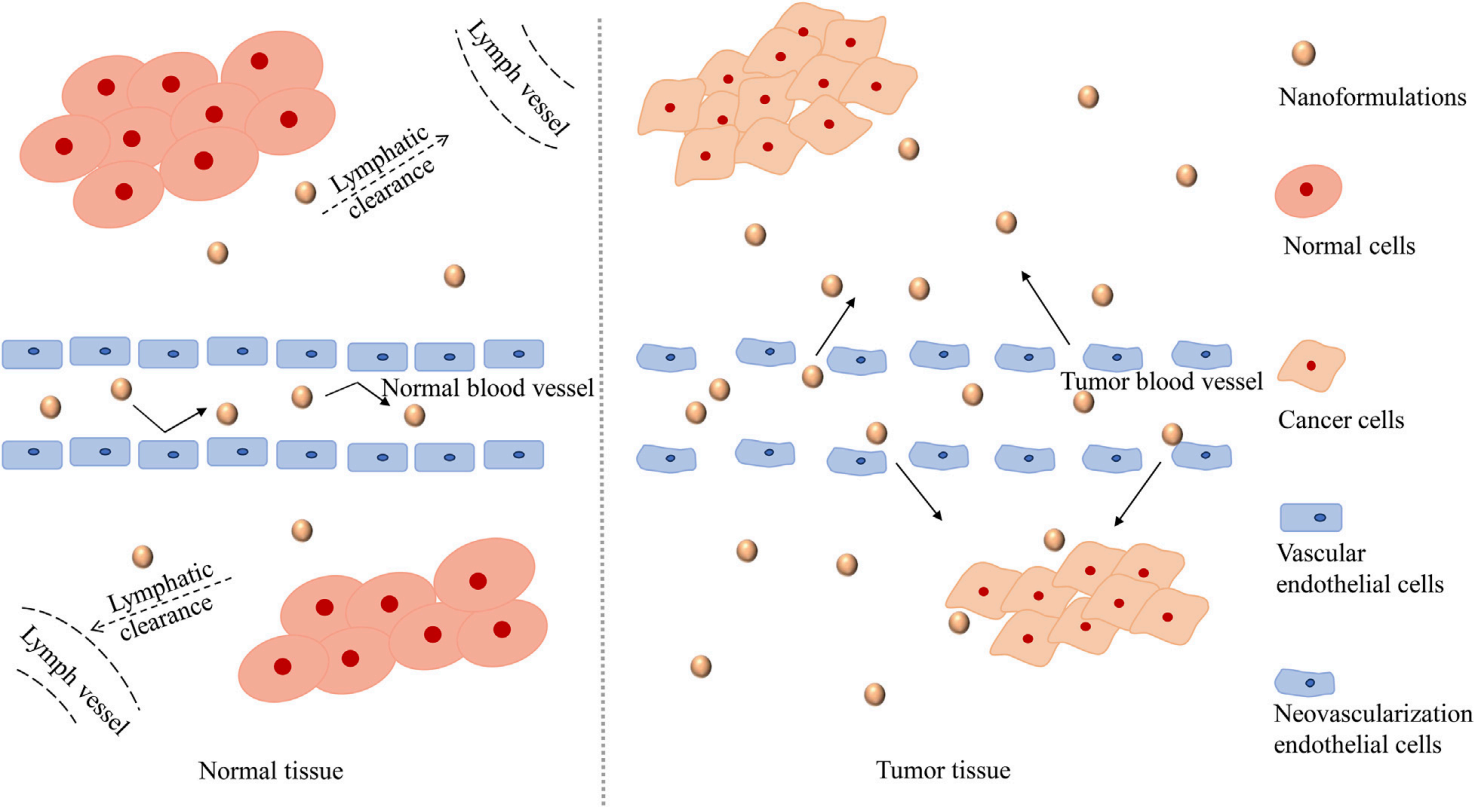
The mechanism of the EPR effect. The EPR effect, which refers to the high permeability of tumor blood vessels and the absence of lymphatic reflux, promotes a high accumulation of nanoformulations in tumor tissue (compared to normal tissue). Due to the rapid growth of tumor cells, the vascular endothelium of tumor tissue is damaged. The vascular endothelium gaps in tumor tissue are wider than that in normal tissue, and thus has higher permeability, which is conducive to the entry of nanoformulations into tumor tissue. Moreover, the lymphatic system in the tumor tissue is damaged and cannot play an effective filtering and clearing role as the normal lymphatic system, so that the nanoformulations are retained in the vicinity of the tumor and released slowly, which improves the targeting performance of nanoformulations. Abbreviations: The EPR effect, the enhanced permeability and retention effect.

Nanoformulations have broader prospects in the field of anticancer research, with the successful marketing of doxorubicin long-cycle liposomes and paclitaxel albumin nanoparticles. The enhanced permeability and retention effect (EPR effect, Figure 3) is the most basic strategy of nanopreparation applied to tumor tissues (Nakamura et al., 2016). The vascular networks of tumor tissues differ greatly from those of normal tissues. The rapid growth of cancer cells incurs a high oxygen demand, leading to angiogenesis in tumor tissues. The new blood vessels are characterized by irregularity and absence of a basement membrane, which leads to the high permeability of the tumor blood vessel network to some extent. In addition, damage to the lymphatic system in tumor tissues inhibits the re-entry of components into blood circulation, thus allowing them to remain in tumor tissues. The EPR effect promotes the high accumulation of nanoformulations in tumor tissues (compared to normal tissues), reduces the side effects of chemotherapy drugs, and provides a good opportunity for the treatment of cancer with nanoformulations (Li Q. et al., 2019; Kalyane et al., 2019).

To date, a variety of UA nanoformulations, such as liposomes, nanoparticles, and micelles, which have effectively improved the pharmacokinetic properties of UA and can act better on tumor tissues through the EPR effect, have been developed successively. In this paper, we summarized the current pharmacokinetic studies on UA, on topics such as the absorption, distribution, and elimination of UA as well as on the developed UA nanoformulations, in an effort to provide references and information for the drug design and rational application of UA.

## Review of UA Pharmacokinetics

### Absorption

As UA is mainly absorbed by the intestinal tract through passive diffusion, its absorption rate is very fast. The concentration of UA reached a peak of 1.10 ± 0.31 µg/mL approximately 0.5 h after the oral administration of 10 mg/kg UA (extracted from *Calendula officinalis Sieb. et Zucc*) (Chen et al., 2011). One study showed that the uptake of UA by Caco2 cell monolayers showed a linear increase, with no obvious saturation trend in the concentration range of 10–40 µg/mL (Jinhua, 2019). Another study reported that the uptake of UA (the suspension dissolved in DEMSO) by Caco-2 cell monolayers was significantly directional. It was also observed that the absorption of UA is influenced by temperature and pH values. The absorption of UA is more active at 4°C (when the temperature of the culture system was 4, 25, and 37°C) and the absorption significantly decreased under alkaline conditions (when the pH value of the culture system was 5.5–8.0) (Hua et al., 2012). The absorption of UA is mainly mediated by P-glycoprotein (P-gp) transporters, and studies have shown that pregnane X receptor (PXR) affects P-gp abundance. After PXR was silenced in Caco2–siRNA–PXR cells, the apparent permeability ratio values were 1.85 ± 0.36, 1.24 ± 0.11, and 1.19 ± 0.04 when UA concentrations were 10, 20, and 50 μM, respectively. In Caco2 cells without PXR silencing, the apparent permeability ratio values increased to 2.19 ± 0.44, 1.40 ± 0.17, and 1.25 ± 0.07, respectively (Jinhua et al., 2020). UA contains hydroxyl (C3) molecules, which are likely to be acidified by glucosaldehydes or sulfates in intestinal cells. After a *β*-glucuronidase/sulfatase treatment, the apparent permeability coefficient (PAPP) of UA in Caco2 cell monolayers decreases from 2.7 × 10^−6^ to 2.3 × 10^−6^ cm/s. Therefore, to some extent, UA acidification or sulfurization results in low UA bioavailability (Qiang et al., 2011).

### Distribution

The UA-related tissue distribution has been studied. In animal models, UA is mainly distributed in the liver, heart, spleen, kidneys, and other organs. After 19.69 mg/kg of UA (extracted from *Hedyotis diffusa Willd*) was administered to healthy Sprague-Dawley rats, organ samples were collected. The tissue concentrations of UA were measured in the liver, kidneys, spleen, heart, and lungs. In addition, the distribution of UA in C57BL/6 mice was analyzed, and the results also showed that the concentrations of UA in the liver, kidneys, and heart increased gradually with the observation time (Yin et al., 2012; Chen X. et al., 2018). These results suggest that UA distribution may be related to blood flow and the perfusion rate. Why is the concentration of UA in the liver so high? Organic anionic transport polypeptides (OATPs) are transmembrane proteins involved in the uptake of various endogenous and exogenous compounds and in the pharmacokinetics and drug-drug interactions of clinically relevant compounds (Patik et al., 2015). Moreover, UA was found to be the substrate of OATP1B1, and OATP1B1 presumably mediates the transport of UA to liver cells, which further explains the phenomenon of high UA concentrations in the liver. Of course, OATP1B3 may also mediate the transport of UA to hepatocytes (Roth et al., 2011).

### Elimination (Metabolism and Excretion)

Liver is an important metabolic organ, in which UA mainly distributed among previous studies. PXR is highly expressed in hepatocytes and plays a key role in regulating some metabolic enzymes in downstream regions. Cytochrome P450 (CYP) enzymes are among the most important metabolic enzymes in the liver. Thus, in the experiments on Caco2 cells and Caco2-PXR-RXRα cells, the Km values of UA are 81.99 ± 44.32 and 60.05 ± 29.62 g/mL, respectively, and the *V*_max_ values are 3.77 ± 0.86 and 3.41 ± 0.96 μg ml^−1^ min^−1^, respectively (Jinhua et al., 2020). In addition, it has also been reported that the conversion rate of UA in human liver microsomes containing the prototype coenzyme II (NADPH) is approximately 40% after a 20-min culture, but no significant conversion has been observed in the absence of NADPH (Qin and Wang, 2019). A pharmacodynamic test showed that after administering 19.69 mg/kg UA to healthy Sprague-Dawley rats, some kinetic parameters measured as follows: apparent volume of distribution (V/F 0.0169 ± 0.0030 L/g), half-life time distribution (*t*_1/2α_ 6.95 ± 1.42 h), half-life time elimination (*t*_1/2β_ 40.94 ± 4.91 h), and maximum plasma concentration (*C*_max_ 487.47 ± 138.94 ng/ml) (Chen X. et al., 2018). These results could be related to molecular proteins that affect UA metabolism. Studies have shown that 611 molecular proteins may interact with UA, and more than 49 functional clusters respond to UA (He et al., 2015). Experiments have shown that, compared with other small molecules, UA has a relatively lower Log*P* value and better lipophilicity, thus it has a better chance to reach the receptor site. Lipophilicity is one of the major determinants of a compound's metabolic properties *in vivo*, which usually estimated by Log*P* (Kalani et al., 2012). In the study of pharmacokinetics, elimination includes two parts: metabolism and excretion. However, there are a few studies on UA excretion. Due to the undetectable UA concentration in urine in some extent, which is below the lower limit for quantitative determination (Jinhua, 2019).

However, it is worth noting that owing to the low solubility of UA, some residues remain in the digestive tract after oral administration, and the intestinal microflora may metabolize UA. The intestinal tract has a special microbiome, which is different from the physiological environment of the liver. Therefore, the metabolic transformation process of UA in the intestinal tract is specific and worthy of further study. The results of the study on UA and intestinal flora showed that UA could reduce the dosage of antimicrobials by 8 times (Dwivedi et al., 2015). UA has a good regulatory effect on intestinal microflora; it can regulate the intestinal microbial community of hamsters and promote the growth of short-chain fatty acid-producing bacteria in the intestinal tract (Hao et al., 2020).

In summary, UA is a hydrophobic drug with poor solubility and oral bioavailability. Hence, it is difficult to exert significant cytotoxic activity. UA is absorbed rapidly by the intestinal tract mainly through passive diffusion, and is also affected by exogenous protein P-glycoprotein or *β*-glucuronidase or sulfate esterase, resulting in poor absorbed blood content. UA is mainly distributed in liver after absorption, which may be related to the transport mediated by OATPs. The concentration of UA in the liver and kidney is increased in a time dependent manner, which may be related to blood perfusion. The metabolism of UA in liver is closely related to the enzyme activity of CYP and NADPH. According to existing studies, we know that only a very small amount of UA is eliminated by renal excretion. Up to now, the pharmacokinetic study of UA still needs further exploration.

## Review of UA Nanoformulations

As the gastrointestinal mucosa permeability of UA is poor and its oral absorption rate is low, the UA-related nanoformulations are designed so as to be administered mainly intravenously to prevent the gastrointestinal absorption process of UA and improve the drug delivery efficiency to the site of action. The basic characteristics and related anticancer research results of UA nanoformulations are shown in Table 2. As was mentioned earlier, nanoformulations selectively accumulate in tumor tissues owing to the EPR effect. Particle size, surface performance, and stability are the three key characteristics of nanoformulations, which affect the accumulation of nanoformulations in tumor tissues (Israel, 2018). As far as the particle size is concerned, it is intended to facilitate the longer blood circulation duration and tumor enrichment of the nanomedicine, while slowing down the excretion from the kidney. Moreover, by modifying surface performance, nanoformulations can be able to evade the immune system, while circulating in the blood system to obtain long circulation performance, so as to increase their chances of flowing through tumor blood vessels and infiltrating into tumor tissues owing to the EPR effect. Regarding stability, in addition to the active drug release required to function in cells, drug molecules must be stably contained in the carrier for the rest of the time so as not to release to the circulatory system or non-target organs (Nakamura et al., 2015; Nakamura et al., 2016; Kalyane et al., 2019).

**TABLE 2:** The preparation, characteristics, and results about the anti-cancer effects of UA nanoformulations.

Type	Preparation	Materials or modified	Particle size (nm)	Zeta potential (mV)	Polydispe-rsity index	Experimental models	Main outcomes	References
Polymer micelles	The thin-film dispersion method	mPEG-PLA	29.35 ± 0.38	0.75 ± 1.30	0.299 ± 0.005	HepG2 cells	Inhibit the proliferation and migration of HepG2 cells; (The IC_50_ values of free UA and UA-PMs at 24 h were 43.2 ± 5.01 and 37.28 ± 2.44 μmol/L, respectively.)	Zhou et al. (2019)
						L-02 cells,	Regulate the growth of L-02 cells, bidirectionally	
						H22 cells implanted tumor xenograft in male Kunming mice	Inhibit the growth of H22 xenograft and prolong the survival time of tumor-bearing mice. (The tumor inhibition rate was 61.43%, and the survival time was increased to 45.6 ± 10.0 days, with 100 mg/kg UA-PMs, respectively.)	
	Self-assemble method	The LMWH–UA conjugate	200–250	–	–	B16F10 cells	Enhance neutralizing effect on angiogenic growth factors	Cheng W. et al. (2017)
						Subcutaneously implanted B16F10 cells tumor xenografts in female C57BL/6 mice	Retard tumor growth and prevent recurrences without risk of hemorrhage. (The inhibition ratios of LMWH–UA, LMWH + UA and LMWH were 41.89, 30.11 and 26.28%, respectively.)	
Liposomes	The ethanol injection method	PEG-modified	100–200	–	–	EC-304 cells	Significantly extend the circulation time	Zhao et al. (2015)
							Possess higher stability and slower release rate than conventional liposomes	
							Possess relatively low cytotoxic effect than UA conventional liposomes within 24 h (71.2 6% vs. 68.27%)	
	The lipid hydration method	Long-circulating and pH-sensitive liposome	191.1 ± 6.4	1.2 ± 1.4	–	MDA-MB-231 cells, LNCaP cells	Significantly inhibit cancer cell proliferation. (The IC_50_ values of free UA and SPHL 20UA1 on MDA-MB-231 cells were 13.07 ± 1.54 and 8.13 ± 2.3 μmol/L respectively. However, there was no statistical significance for LNCaP cells.)	Caldeira de Araújo Lopes et al. (2013)
	The lipid hydration method	Long-circulating and pH-sensitive liposome	182.7 ± 8.0	0.3 ± 1.6	0.60 ± 0.07	MCF-7 tumor-bearing mice	Seem to induce an antiangiogenic effect in the human breast tumor model	Rocha et al. (2016)
							Tumor growth inhibition was not observed in human breast tumor–bearing animals	
	The thin-film dispersed hydration method	FA-modified	160.1 ± 12.5	21.24 ± 4.2	0.196 ± 0.052	KB cells, Subcutaneously implanted KB cells tumor xenografts in female Balb/c nu/nu mouse	Induce more cytotoxicity and higher apoptosis	Yang et al. (2014)
							Significantly higher human epidermoid carcinoma (KB) inhibition. (The IC_50_ values of FTL-UA and FR blocking group were 22.05 and 65.66 µM, respectively.)	
	The thin-film dispersed hydration method	FA-modified	165.1	18.6	–	KB cells	Significant inhibition of cell growth; (The inhibition rate was 71%.)	Li W. et al. (2019)
		Bmi1 siRNA				Human KB tumor xenograft nude mice	Significant positive correlation between Bmi1siRNA and UA co-delivered by folate-targeted liposomes to inhibit tumor cells and revealed enhanced cytotoxic effects (The tumor volumes of normal saline group, free UA group, UA-L group, FA-UA-L group, and F-UA/siRNA-L group were 2,254, 1,300, 1,156, 753, and 318 mm^3^, respectively.)	
	The ethanol injection method	CS-modified	135.4 ± 0.636	7.8	0.2	HeLa cells	Reduce the drug dosage and side effects	Wang M. et al. (2017)
						Mice bearing U14 cervical cancer	Exhibit obvious anti-proliferative effect (76.46% on HeLa cells) and significantly antitumor activity	
							Enhanced cell apoptosis, extensive necrosis and low cell proliferation activity (61.26% in mice bearing U14 cervical cancer)	
	The ethanol injection method	PLL, HA	102.0 ± 3.0	−8.5 ± 1.1	0.254 ± 0.028	SCC-7 cells	Achieve the programmed apoptosis for anticancer action	Poudel et al. (2020)
						BT-474 cells	Exhibit pronounced anticancer effect. (In SCC-7 cells, the cell viabilities treated with UA and UA-PLL-HA.P at 48 h were about 41 and 21%, respectively; under the same conditions, in BT-474 cells, the cell viabilities were about 40 and 33%, respectively.)	
Nanoemulsion	Mechanical method	OA, UA	198.95 ± 21.34	–	0.285 ± 0.053	B-16 cells	Significantly high antioxidant (>85%) and anti-cancer activity (The cytotoxic activity of the compound decreased from 17.4 to 2.9 µM after making nanoemulsion.)	Alvarado et al. (2018)
Nanocrystals	The anti-solvent precipitation method	UA	188.0 ± 4.4	−25.0 ± 5.9	0.154 ± 0.022	MCF-7 cells	Showed good aqueous dispensability and a higher dissolution rate	Song et al. (2014)
							Significantly induce stronger cell growth inhibition activity by inducing G2/M phase cell cycle arrest. (The IC_50_ values of free UA and UA nanocrystals at 24 h were 15.42 ± 1.19 and 7.90 ± 1.11 μmol/L, respectively.)	
	The high pressure homogenization method	UA	291.7 ± 7.5	−14.0 ± 1.9	0.260 ± 0.021	Sprague-Dawley rats	a significant increase was observed in the dissolution rate of UA nanocrystals	Pi et al. (2016)
							The relative bioavailability of UA nanocrystals exhibited 2.56-fold enhancement than that of UA coarse suspension	
	The antisolvent precipitation method	UA	101.2 ± 3.53	−9.79 ± 0.794	0.205 ± 0.012	MCF-7 cells	Compared with the UA solution-treated cells, the population of MCF-7 cells in the early and late apoptotic phases was increased respectively by 49 and 52% when treated with the 100 nm nanosuspension and 82 and 69% when treated with the 300 nm nanosuspension	Wang et al. (2015)
			299.8 ± 6.63	−8.19 ± 0.782	0.150 ± 0.021			
Nanoparticles	Nano-precipitation method	CS	100–200	41.6	0.08	HUVECs	Inhibit the proliferation, migration, and tube formation of HUVECs; (The IC_50_ of UA and CH-UA-NPs was 82.5 and 56.7 μg/ml, respectively.)	Jin et al. (2016b)
						CAM	Reduce the angiogenesis in CAM of fertilized eggs; (CH-UA-NPs at 2μg/CAM could effectively reduce angiogenesis as compared with that of control group.)	
						BALB/c mice vaccinating ascites mouse H22 cells	Inhibit the H22 tumor growth through anti-angiogenesis induced by VEGF signaling pathway blocking. (The volume of tumors of nanoparticle-treated group and control group was 1.12 ± 0.12 and 2.36 ± 0.32 cm^3^)	
	Nano-precipitation method	FA-modified, CS	160	39.3	–	MCF-7 cells	Effectively diminish off-target effects and increase local drug concentrations of UA	Jin et al. (2016a)
						MCF-7 xenograft mouse model	Induce overproduction of ROS and destruction of mitochondrial membrane potential, and resulted in the irreversible apoptosis in cancer cells. (The tumor weight of FA-CS-UA-NPS group, UA group and normal saline control group was 2.1 ± 1.02 g, 3.48 ± 0.24 g and 5.26 ± 1.69g, respectively.)	
	Single-emulsion solvent evaporation technique	PLA	246 ± 10	−24.6 ± 3.1	0.148 ± 0.014	B16-F10 cells	Reduce the cell viability in 70% after 72 h	Antônio et al. (2017)
	Nano-precipitation method	PVP-b-PCL	120.0 ± 4.0	−0.96 ± 0.3	–	H22 cells, Subcutaneously implanted H22 cells tumor xenografts in male ICR mice	Inhibit the growth of liver cancer cells and induced cellular apoptosis more efficiently than did free UA; (IC_50_, 32.89 ± 3.23 µM vs. 59.84 ± 4.12 µM; CT findings confirmed that the tumor lesions in the UA-NPS group showed near total depletion.)	Zhang et al. (2015)
							Significantly delayed tumor growth	
							More significant effect on protein expression than did free UA	
							Upregulated the expressions of Caspase-3 and Bax, but downregulated the expressions of Bcl-2 and COX-2	
	Single-emulsion solvent evaporation technique	PLGA	154 ± 4.56	−18.4	0.29	B16-F10 cells	Exhibit slower blood clearance and comparatively high uptake in tumor region	Baishya et al. (2016)
							Exhibit dose-dependent activity in comparison to free drug. (The IC_50_ values of free UA and UA-NPs were 60 and 18 μM respectively following 48 h incubation.)	
	−	MSNs	102.2 ± 6.5 (PH = 10)	–	–	HepG2 cells	Exhibit sustained release profile in the initial 20 h	Li T. et al. (2017
							Exhibit higher proliferation inhibition, cell cycle arrest at the G2/M phase and significantly caused the early and late apoptosis in HepG2 cells. (The early and late apoptosis rates of HepG2 cells treated with control group, UA, and UA@MSN-UA were (2.74, 1.89%), (4.77, 4.72%), and (11.7, 19.4%), respectively.)	
	–	MSN-CS-LA	197.7 ± 3.5	6.3 ± 1.4	0.368 ± 0.027	SMMC-7721 cells, Subcutaneously implanted H22 cells tumor xenografts in male Kunming mice, H22 lung metastasis models	Exhibit pH-responsive function and sustained release profile	Zhao et al. (2017)
							attenuate the adhesion, migration of ASGPR over-expressing liver cancer SMMC-7721 cells	
							Significantly increased the cellular apoptosis and down-regulated the expression of EGFR and VEGFR2 proteins; (In the SMMC-7721 cells, the IC_50_ for UA, UA@MSN-COOH, and UA@MSN-CS-LA group was 24.97, 21.99, 18.25 mM, respectively.)	
							Reduce the tumor burden; inhibit the lung metastasis	
	−	MSN-FA	209 ± 9.21	−12.2 ± 1.35	0.23 ± 0.07	HepG2 cells	Observe that 80% of free UA was released within 10 h	Zheng et al. (2017)
						HeLa cells	Show a sustained release profile in release media; improve the antitumor effect	
	Self-assembly approach	UA, PXT, IGG	130.8 ± 0.20	−30.0 ± 0.80	0.117 ± 0.003	HeLa cells, HepG2 cells, H22 cells, Kunming mice with subcutaneous H22 cells xenografts	Significantly improve water solubility and bioavailability of UA	Guo et al. (2017)
							Remarkably inhibit the viability of cancer cells under NIR laser irradiation; (The 21 days survival rate of the mice models was 100%, the tumor inhibitory rate was 89.18 ± 1.19%, and no tumor recurrence was detected.)	
							Possess imaging function and exhibited effective passive tumor targeting to tumor site	
	Self-assembled method	LA, IGG	116.4 ± 2.4	−30.6 ± 1.8	0.201 ± 0.02	HepG2 cells, HeLa cells, H22 cells, Murine H22 hepatocarcinoma tumor-bearing model	Exhibit significant targeting to HepG2 cells due to the presence of ASGPR and EPR effect	Zhao et al. (2018)
							Present a notable anti-proliferative activity on the ASGPR-overexpressing HepG2 cells than ASGPR low-expressing HeLa cells	
							Display remarkable antitumor activity in H22 xenograft mice. (The tumor inhibition rate of UA-LA-ICG NPs + NIR was up to 96.32% compared with the control group.)	

*Abbreviations* ASGPR, asialoglycoprotein receptor; Bcl-2, B-cell lymphoma-2; Bax, Bcl-2-associated X protein; CAM, chicken chorioallantoic membrane; COX, cyclooxygenase; CS, chitosan; EGFR, epidermal growth factor receptor; EPR, enhanced permeability and retention effect; FA, folate; HA, hyaluronic acid; HUVECs, human umbilical vascular endothelial cells; IGG, indocyanine green; LA, lactobionic acid; LMWH, low molecular weight heparin; mPEG, methoxy poly (ethylene glycol); MSN, mesoporous silica nanoparticle; NIR, near-infrared; OA, oleanolic acid; PCL, poly (ε-caprolactone); PLA, poly(l-lactic acid); PLGA, Polylactic-co-glycolic acid; PLL, poly-l-lysine; PVP, poly(N-vinylpyrrolidone); PXT, paclitaxel; ROS, reactive oxygen species; UA, ursolic acid; VEGF, vascular endothelial growth factor; VEGFR, vascular endothelial growth factor receptor.

It should be noted that the enrichment of nanoformulations into tumor tissues is inevitable through the tumor microenvironment, where the pH value is more acidic than that of normal tissues, and tumor tissues and their cells contain higher concentrations of glutathione (GSH), reactive oxygen species (ROS), and highly expressed metabolic enzymes (Zhang et al., 2020b). Thus, it could be suggested that the design of nanoformulations should enable them to change their nanometer size, surface, and stability with changes in the blood system, tumor tissues, and the microenvironment of tumor cells, so as to be able to target tumor tissues and reach them efficiently. The property conversion of the nanoformulations can be realized by properties containing many of the same or different functional groups (Schmidt et al., 2018; Cuevas-Flores et al., 2020).

### Polymer Micelles of UA

Polymer micelles are new nanocarriers formed by the self-assembly of copolymers in aqueous solutions. They are usually amphiphilic and have a nucleus-shell structure with a hydrophobic core and a hydrophilic shell (Masserini, 2013; Du and Chen, 2019). The hydrophobic core of micelles can contain hydrophobic antitumor drugs and can increase the solubility and stability of the drugs. The hydrophilic shell can prevent the phagocytosis of the reticuloendothelial system (RES) and keep it stable in the blood circulation for a long time (Li Z. et al., 2018; Ma et al., 2019).

Zhou et al. prepared a UA-loaded polymer micellar delivery system (UA-PMs) using mPEG-PLA (PEG hydrophilic, PLA hydrophobic) by membrane dispersion, and investigated its the proliferation inhibition effect on HepG2 (human liver cancer cells), L-02 (human normal liver cells), and H22 (mouse liver cancer cells) cell lines. The ratio of UA to the carrier was approximately 1:10, and the micelle was smooth and spherical. The average particle size, zeta potential, polymer dispersion coefficient, and the critical micelle concentration was 29.35 ± 0.38 nm, −0.75 ± 1.30 mV, 0.299 ± 0.005, and 2.3 × 10^−3^ mg/mL, respectively, which showed good micelle stability and increased circulation time in the blood. In addition, the release of the UA-PMs at pH 7.4 and 5.5 was evaluated at 37°C owing to the microenvironment of the tumor tissues with microacidic properties. The results showed that the UA release of the UA-PMs was significantly comparable (pH = 5.5, 24 h, 65% vs. pH = 7.4, 96 h, 50%) and there was no obvious initial outburst, indicating that the micelles had controlled release behavior and could increase the accumulation of UA at tumor sites to some extent. This could be because UA is protected from degradation by the polymer micellar hydrophobic core. In addition, compared with free UA, the proliferation and migration ability of HepG2 cells are reduced to a certain extent with the increase in the UA-PM concentration. Moreover, neither free UA nor UA-PMs are significantly toxic to L-02 cell lines. Interestingly, UA and UA-PMs promote L-02 cell proliferation at low concentrations (approximately <60 um). In animal experiments, UA-PMs showed stronger tumor inhibition than free UA at the same concentration; additionally, they showed a concentration-dependent tumor inhibition rate (Zhou et al., 2019).

To improve the controllability and tumor targeting of polymer micelles, it is necessary to make them more sensitive to microenvironmental changes. Therefore, research on polymer micelles has been undertaken in multiple directions (Chen et al., 2014). Different ligands or related antibodies can be modified on the surface of polymer micelles, or materials can be used to make the micelles sense changes in the microenvironment, such as, pH and oxidative stress *in vivo*, so as to meet the requirements of targeted and controlled release (Zhang et al., 2016).

### UA Liposomes

Liposomes are vesicles composed of one or more lipid bilayers layers, first discovered by British scientist Bangham in the 1960s (Su et al., 2018). Liposomes can carry both hydrophilic (embedded in the lumen of a liposome) and hydrophobic substances (embedded in a lipid hydrophobic bilayer) (Ghadiri et al., 2019). Liposomes have been used for drug delivery since 1971; compared with other nanodelivery systems, liposomes have certain advantages, such as good biocompatibility and degradability as well as low immunogenicity. This constitutes liposomes promising for application in drug delivery (Yang et al., 2017). At present, liposomes have been modified from their initial, classical lipid composition, to develop long-circulating, environmentally sensitive, and actively targeted liposomes; through organic combinations, a multi-functional liposome that has higher clinical application potential has been obtained.

#### Long Circulating Liposomes

Liposomes are composed of dynamic phospholipid membranes, which have thermal instability. Moreover, the destruction of proteins and enzymes in the blood environment is not conducive to the stability of the liposome membrane, leading to its rupture and the leakage of the encapsulated drugs (Yan et al., 2016; Tanka-Salamon et al., 2017). In addition, the mononuclear macrophage system (MPS) recognizes and ingests liposomes to reduce the amount of drugs reaching the target site (Martinez et al., 2018). In order to solve the aforementioned problems, polyethylene glycol (PEG)-modified liposomes with good effect and marketization have been employed. PEG molecules can form a hydration film on the surface of liposomes, which is helpful in increasing the resistance and improving the stability of liposomes. In addition, PEG molecules block the interaction between positive charge and proteins, reduce the probability of being recognized and absorbed by MPS, and significantly lengthen the circulation time of liposomes in the body. Therefore, they are called long circulating liposomes (Zeng et al., 2016; Yuba et al., 2018).

It has been confirmed that PEG-modified UA liposomes have higher stability and slower release rates compared with ordinary liposomes. In one study, the ratios of the components of PEGylated UA liposomes were determined; the UA, PEG, cholesterol, and soy lecithin ratio was 3:2:5:30. The UA liposomes showed a regular uniform spherical shape with good dispersion and average particle size between 100 and 200 nm. In addition, after PEGylation, UA liposomes were harder than ordinary liposomes, which, to some extent, prevented them from collapsing in space, further improved their membrane stability, and prevented the sudden release of loading drugs. After a 17-days observation at 25°C, the encapsulation rates of the modified liposomes and the ordinary liposomes were 63.18 and 55.18%, respectively. Moreover, the modified liposomes could circulate in the blood for 48 h, significantly extending the circulation time and increasing the drug content at the targeted sites. It should be noted that in subsequent *in vitro* anti-tumor experiments, the modified liposomes did not show a higher tumor inhibition rate than the normal liposomes (68.27 vs. 71.26%). This may be related to the slow release of liposome drugs after modification, and the increased observation time (24 h of cell culture *in vitro* in this study), which may yield opposite tumor suppressive results (Zhao et al., 2015).

#### pH Sensitive Liposomes

In one study, the pH value of the extracellular microenvironment of the tumor was between 6.5 and 7.2, which was more acidic than that of normal tissues (∼7.4), while the intracellular lysosome pH was lower than 6.0 (Hu et al., 2016). This step change in pH in tumor tissues has attracted the attention of researchers and led to the design and synthesis of pH-sensitive liposomes based on long-circulating liposomes. The pH-sensitive liposomes are stable under physiological conditions and break the connection bonds when located in an acidic environment, thereby triggering the release of drugs and increasing their release rate in specific tissues as well as improving the targeting of tumor tissues by the drug (Chen et al., 2013). However, it is worth mentioning that researchers have obtained inconsistent results in preclinical studies of breast cancer regarding pH-sensitive liposomes; therefore, further studies are required. Oliveira et al., successfully prepared a long-circulating pH-sensitive liposome (SpHL-UA) with an average particle size of approximately 191.1 nm, which exhibited significant inhibition of MDA-MB-231 cell lines in breast cancer (SpHL-UA, IC_50_ 8.13 µM vs. free UA, IC_50_ 13.07 µM) (Caldeira de Araújo Lopes et al., 2013). However, in another study, tumor growth inhibition was not observed in MCF-7 tumor-bearing mice treated with SpHL-UA (the particle size was approximately 182.7 nm) (5 days), but anti-angiogenesis was observed to a certain extent (Rocha et al., 2016). This could have been due to the different models employed or the reduced liposome action time, which may have resulted in absent treatment effects during the observation period.

#### Actively Targeted Liposomes

Actively targeted liposomes are prepared by adding some targeting materials to liposomes, which can further increase the accumulation of drugs in tumor tissues. Folic acid (FA) receptors are widely used in targeted delivery systems because of their high expression in many cancer cells and low expression in normal tissues, as well as the high affinity between FA molecules and FA receptors or folate-binding proteins expressed in cell membranes (Yi, 2016; Zhang et al., 2017). By using ligand-receptor specific binding, the anti-cancer UA has been successfully delivered to tumor cells through effective tumor targeting mediated by FA receptors.

One study showed that the average particle size of the folate-targeted UA liposomes was 155 nm, with good stability and nearly 31-fold (compared with the non-targeted liposomes) drug delivery efficiency. Subsequent anti-cancer studies have produced better results. Folate-targeted UA liposomes significantly reduce the tumor volume of tumor-bearing mice (by approximately 55%), and their IC_50_ is significantly lower than that of non-targeted liposomes (22.05 vs. 146.3 µM). Moreover, the cytotoxicity of folate-targeted UA liposomes induced by apoptosis is significantly dose- and time-dependent (Yang et al., 2014). In another study targeting UA liposomes with FA, Bmi1 siRNA (the gene encoding polycomb repressive complex 1; small interfering RNA) was adsorbed on the surface of liposomes in addition to planting FA molecules. The downregulation of Bmi1 inhibits tumor cell growth in different cancer types, and siRNA can downregulate the expression of oncogenes. Finally, a uniformly stable liposome FA-UA/siRNA-L with an average particle size of approximately 165.1 nm was successfully prepared. After a 24-h FA-UA/siRNA-L treatment, the inhibition rate of KB cells (human oral cancer cell line) was 71%. In an antitumor tumor-burdened mice observation experiment, the tumor sizes of the normal saline, free UA, UA liposome, UA folate liposome targeting, and FA-UA/siRNA-L groups were 2,254, 1,300, 1,156, 753, and 318 mm^3^, respectively, which suggests that FA targeted liposomes can significantly enhance the UA anti-cancer effect, and UA and Bmi1 siRNA have some synergistic antitumor effects (Li W. et al., 2019).

In addition to FA molecules, glycosyl-specific binding receptors are also highly expressed in the cell membrane of human tissues. Therefore, it would be worth considering to add polysaccharide-modifying components, such as hyaluronic acid (HA) molecules, in the liposome structure to improve targeting. Sun et al., successfully prepared UA containing nanoliposomes encapsulated in hyaluronic acid, which showed better anti-liver cancer activity than 5-fluorouracil. However, it should be mentioned that UA was not the only drug targeting tissues in this study; ginsenoside and oleanolic acid (OA) were used as well (Sun S. et al., 2020).

#### Multifunctional Liposomes

It is worth noting that the tumor microenvironment is complex and changeable, and there may be pH value, enzyme activity, and ROS content differences overall or depending on the layer. Therefore, a single modification cannot fully cope with the microenvironment and all the accompanying changes. Therefore, in order to achieve higher transmission efficiency and better therapeutic effects, multifunctional liposomes with reasonable superposition of different modification modes have been developed by scientists.

Poudel et al. successfully prepared a new UA nanoliposome with dual pH and enzyme response through the use of poly-l-lysine (PLL) and HA in recent years. This nanoliposome can enable drugs to locate tumor sites more accurately and improve their bioavailability at target sites. PLL is a cation that is pH-responsive by protonation. HA is an anion that reacts with hyaluronidase (HYAL) in tumor tissues and enters cells by binding specifically to CD44 receptors. In the aforementioned study, cholesterol (Chl), phosphatidylcholine (PC), and UA were prepared into UA liposomes (UA.P) at a ratio of 1:2:0.5. The particle size and zeta potential were 85.7 ± 1.5 nm and −19.3 ± 0.2 mV, respectively. On this basis, UA-PLL.P was constructed by coating the PLL layer with a liposome with a particle size of 91.3 ± 1.1 nm and zeta potential of 32.6 ± 0.2 mV. Finally, the HA layer was coated with a liposome with a particle size and zeta potential of 102.0 ± 3.0 nm and −8.5 ± 1.1 mV, respectively, to construct UA-PLL-HA.P. The results showed that the sustained release time of UA liposomes was significantly prolonged after surface modification. Additionally, UA-PLL-HA.P had the highest release efficiency of only 38% at 80 h under acidic conditions (pH = 5.0), which was due to the protection of the HA layer. After adding HYAL, the release of UA-PLL-HA.P was approximately 70% within 20 h. Subsequent trials also showed that UA-PLL-HA.P had higher cytotoxicity and better anticancer efficacy than UA.P and UA-PLL.P. When the compound UA and two liposomes, UA.P and UA-PLL-HA.P, which were equivalent to 100 g/mL UA, were applied to SCC-7 cell lines for 24 h, the cell mortality was 45, 51, and 67%, respectively, and increased to 59, 65, and 79% after 48 h, respectively. Similar results were obtained when the same method was applied to BT-474 cell lines. Despite multiple modifications, the UA liposomes in this experiment also exhibited biphasic release. In other words, multiple modifications resulted in higher sustained release efficiency of UA liposomes compared with a single modification, but the sudden release did not seem to be improved (Poudel et al., 2020).

### UA Nanoemulsions

Nanoemulsion (NE) is a type of thermodynamically stable, colloidal dispersion system spontaneously formed by water and oil phases, surfactants, and co-surfactants in proper proportions (Liu et al., 2016). The surfactant can form an adsorption layer at the oil–water interface; this layer can further prevent the aggregation and flocculation of nanoemulsions through electrostatic repulsion and spatial stability, which greatly enhances the stability of nanoemulsions (Khan et al., 2018; Li Z. et al., 2020). In addition, surfactants can increase the solubility of water-insoluble drugs and promote the entry of drugs into small intestinal epithelial cells. This constitutes nanoemulsion a delivery system that can improve the bioavailability of UA and has great potential for sustained drug release and tumor targeting (Liao et al., 2019).

Miastkowska and Śliwa (2020) designed and successfully synthesized a UA nanoemulsion with a particle size of 248 ± 15 nm, but no tests related to biological activity have been conducted. Alvarado et al., successfully prepared a UA-containing nanoemulsion, which exhibited strong proliferation inhibition against B16 melanoma cell lines (the IC_50_ was between 0.58 and 2.9 µM). Unfortunately, this nanoemulsion contains both UA and OA, and UA is not the only active drug. In the UA and OA nanoemulsion system, the average droplet diameter is approximately 198.95 ± 21.34 nm, with good stability and no obvious aggregation or deposition. In addition, in this study it was also observed that the emulsion prepared had a significant antioxidant effect, and the inhibition rate of 2,2-Diphenyl-1-picrylhydrazyl (DPPH), a stable free radical, was approximately 88% (Alvarado et al., 2018). This may also be related to the oil phase of the emulsion being castor oil, in addition to the antioxidant capacity of UA itself, as castor oil has good antioxidant activity (Talukdar et al., 2019). These findings also demonstrate that nanometer emulsions have some advantages over other nanometer delivery systems.

### UA Nanoparticles

Nanoparticles, including (organic) polymer nanoparticles and inorganic nanoparticles, are new drug carriers with great potential for development. The former are generally made using chitosan, polylactic acid, polycaprolactone, and other natural or synthetic polymers, while the latter are generally made using inorganic materials such as carbon, silicon dioxide, and bioceramics (Cheng L. et al., 2017). With respect to anti-tumor therapy, the nanoparticle drug-carrying system is conducive to the high concentration and continuous release of drugs and prolongs the retention time of drugs in the tumor, thereby improving the utilization rate of drugs, slowing tumor growth, and increasing anticancer efficacy (Majidzadeh et al., 2020).

#### Chitosan Nanoparticles

Chitosan is the only alkaline polysaccharide among natural polysaccharides. As a new nanosystem carrier, chitosan has good biocompatibility and biodegradability (Xia et al., 2018; Zhang Y. et al., 2019). UA has good ability to inhibit tumor angiogenesis, but its poor water solubility directly limits its effect.

Jin et al., successfully prepared a UA-loaded chitosan nanoparticle (CH-UA-NPS) with a drug loading rate of approximately 54% and a particle size of approximately 100 nm. The results of both cell and animal experiments showed that CH-UA-NPS has a significant inhibitory effect on angiogenesis. Compared with free UA, the killing effect of nanoparticles on human umbilical vein endothelial cells was more obvious at the same concentration for 24 h (IC_50_:82.5 vs. 56.7 μg/mL). In the mouse hepatocellular carcinoma model, no significant microangiogenesis was observed under the action of CH-UA-NPS; on the contrary, a high amount of tissue necrosis was observed. Cell necrosis may be related to the destruction of lysosomes and the mitochondrial membrane structure owing to CH-UA-NPS entering the cell. The inhibitory effect of CH-UA-NPS on angiogenesis was also observed in a chick embryo chorioallantoic membrane model. Notably, CH-UA-NPS has also been shown to have a good anti-breast cancer effect. FA is a widely used targeting molecule (Jin et al., 2016b).

The successful synthesis of folate-targeting UA chitosan nanoparticles (FA-CS-UA-NPS) has been reported in a study (Jin et al., 2016a). Similar to the UA nanoparticles prepared by Jin et al., FA-CS-UA-NPS also damaged the membrane integrity of lysosomes and mitochondria when they entered the cell, thus inducing the death of cancer cells. The same study also showed that, within a certain range, the uptake of FA-CS-UA-NPS by cancer cells increased with increasing temperature, time, and dose. Cell tests showed that FA-CS-UA-NPS was more lethal than free UA against breast cancer MCF-7 cells. In order to observe the anti-breast cancer animal effect of FA-CS-UA-NPS, the researchers selected female MCF-7 tumor-bearing mice. After a randomized administration, the tumor weight was measured. The values of the FA-CS-UA-NPS, UA, and normal saline control groups were 2.1 ± 1.02, 3.48 ± 0.24, and 5.26 ± 1.69 g, respectively. These results indicated that the ability of FA-CS-UA-NPS to inhibit tumor growth is significant. In addition, experiments showed that FA-CS-UA-NPS may have an immune-boosting effect (superior to that of free UA) (Jin et al., 2016b).

#### Polylactic Acid Nanoparticles

Polylactic acid (PLA), a product of a certain amount of lactic acid condensation reaction, is a biodegradable polymer material with good biocompatibility and is an ideal carrier for drug-carrying nanoparticles (Wang Z et al., 2020d). One study successfully prepared a UA polylactic acid nanoparticle with a high encapsulation rate of up to 96%, which meant that the loss of UA was greatly reduced during the preparation process (Antônio et al., 2017). The cell test confirmed that UA polylactic acid nanoparticles had a certain killing ability against melanoma cells and were highly time-dependent. B16-F10 cell lines were observed under the action of different nanoparticle concentrations for 24 h, and more than 80% of the cells survived; 48 h later, less than 40% of cells survived under high nanoparticle concentrations; 72 h later, the survival of cells was below 50% under all nanoparticle concentrations. This is related to the *in vivo* release process of the nanoparticle, which has the ability of continuous slow release (72 h; 45% release) after an initial sudden release (8 h; 30% release). This suggests that by extending the observation cycle further, we may be able to observe more significant cellular lethality.

#### Polycaprolactone Nanoparticles

Polycaprolactone (PCL) is also a biodegradable polymer material that is chemically synthesized. Zhang et al., successfully prepared UA-containing polycaprolactone nanoparticles (UA-NPs) with an encapsulation rate of over 80%. UA-NPs also exhibited a biphasic release similar to that of the aforementioned PLA nanoparticles, with UA release rates of approximately 28% within 8 h. Both UA and UA-NPS can induce the death of H22 HCC cells, but the latter is more cytotoxic. The IC_50_ of UA and UA-NPS was 59.84 ± 4.12 and 32.89 ± 3.23 µM, respectively. Compared with UA, UA-NPS exhibited stronger tumor suppressive ability in H22 transplanted mice treated with UA and UA-NPS. CT images of the mice showed that UA alleviated most tumor lesions, while the UA-NPS group was almost completely exhausted. Further studies demonstrated that this could be because UA and UA-NPS increased the expression of Bax and Caspase-3 and decreased the expression of Bcl-2, while UA-NPS decreased the expression of Bcl-2 more clearly (Zhang et al., 2015).

#### Poly (Lactic-co-glycolic Acid) Nanoparticles

A Poly (lactic-co-glycolic acid) (PLGA) consists of the random polymerization of two monomers, lactic acid and glycolic acid. It is also a biodegradable functional polymer organic compound with good biocompatibility, good sac formation performance, and good membrane formation (Liu et al., 2020). One study confirmed that PLGA nanoparticles containing UA had a significant concentration-dependent cytotoxicity on B16-F10 mouse melanoma cell lines, with an IC_50_ of 18 μM and a free IC_50_ of 60 μM. Similarly, the nanoparticles exhibited a biphasic release, and approximately 30% of UA was released within the first 4 h (Baishya et al., 2016). Studies have also shown that PLGA nanoparticles containing UA have good application prospects in the treatment of cervical cancer (Wang S. et al., 2017). One study confirmed that the prepared nanoparticles had good proliferation inhibition and significant apoptosis promotion effects on the three cervical cancer cell lines (SiHA, CaSki, and HeLa). Similar results have been reported in animal studies. This may be due to the high expression of P53, increased expression of caspase-3, caspase-8, and caspase-9, and decreased expression of Bcl-2 in cancer cells after nanoparticle treatment. In addition, PLGA nanoparticles containing UA have been shown to have potential therapeutic effects on human retinoblastoma (Silva et al., 2019). In one study, a mixture of UA and OA was used to prepare two kinds of nanoparticles, one loaded with a natural UA/OA mixture, and the other loaded with a synthetic UA/OA mixture. OA is an isomer of UA, which, like UA, belongs to the pentacyclic triterpenes and has significant anticancer properties (Cao et al., 2018). Both nanoparticles were stable (for at least 6 months) and were able to achieve an approximate 75% drug release within 72 h. In this study, the researchers observed the cytotoxicity of the prepared nanoparticles on Y-79 cell lines. The results showed that both nanoparticles had significant cytotoxicity and were concentration-dependent. When treated with 32 umol/L of a natural UA/OA mixture of nanoparticles, approximately 87% of the Y-79 cell lines died.

#### Mesoporous Silica Nanoparticles

Nanoparticles prepared from materials such as chitosan, polylactic acid, and polycaprolactone are biodegradable nanoparticles (Souto et al., 2019). They may undergo hydrolysis or degradation reactions with environmental changes, which may increase unnecessary losses during transportation and ultimately affect the anti-cancer therapeutic effect of nanoparticles. However, Mesoporous silica nanoparticles (MSNs) can not only induce sustained drug release when loaded with more drug molecules, but also protect the drug from degradation by biological enzymes and reduce drug loss during transportation. In addition, studies have shown that the MSN surface is rich in silica hydroxyl, which is easy to modify and enables the design of different functional surfaces to meet different transport needs (Schmid et al., 2016).

The pH-sensitive MSN drug delivery system is a common controlled release system in the MSN drug delivery system, which allows the drug to be released well in acidic tumor tissues. Li et al., successfully prepared pH-sensitive MSNs containing UA. The release test results showed that the UA release rate at pH 5.5 was higher than that at pH 7.4 (60 vs. 40%). In addition, the experiment showed that, compared with free UA, the MSNs infiltrated rapidly HepG2 cells, inhibited significantly the cell cycle, and promoted apoptosis (Li T. et al., 2017). The pH-sensitive MSNs containing UA prepared by Zhao et al. (2017) also achieved good experimental results and showed good drug sustained-release effects. At the same time, they can inhibit the proliferation and migration of SMMC-7721 cells in liver cancer, promote the apoptosis of the cell line, and have significant hepatocellular toxicity. Zheng et al., designed UA-containing MSNs with FA targeting capability. In the study, the authors observed that MSNs showed significant sustained release under the same pH (7.4), and the release rate of UA was maintained at 75% after 48 h. The high uptake of HeLa cells into the prepared nanoparticles was observed by fluorescence labeling. In addition, targeted modification has been shown to significantly improve the toxic effect of MSNs on HeLa cells with high FA expression (Zheng et al., 2017).

### UA Nanocrystals

Nanocrystals (also known as nanocrystal suspensions or nanosuspensions) usually refer to the nanoscale dispersion system of pure drugs dispersed in liquid in crystalline or amorphous form, with a small amount of either a surfactant or polymer as a stabilizer (Teymouri Rad et al., 2017; Vora et al., 2018). Generally, nanocrystals do not use carriers, have few excipients, are simple to prepare, and have a high drug load. If the drug nanocrystals are controlled by particle size or modified by surface, they can also target specific parts, such as the liver, spleen, brain, or tumor tissues (Qin et al., 2018). In addition, studies have shown that nanocrystals can improve the uptake of insoluble drugs in Caco-2 cells and the transmembrane transport rate (Di Costanzo and Angelico, 2019). These findings resulted in a new method to increase UA dissolution, improve UA bioavailability, and constitute the anticancer activity of UA more efficient.

In one study, UA nanocrystals with a particle size of approximately 188.0 nm were prepared successfully, with a dissolution rate of approximately 100% within 2 h in 0.5% sodium dodecyl sulfate solution. Subsequent experiments showed that UA nanocrystals had concentration-time dependent cytotoxic effects on MCF-7 cell lines of breast cancer, with an IC_50_ value of approximately half that of free UA. Generally speaking, the smaller the nanocrystal particle size, the faster the drug is dissolved, and thus, better absorption may be achieved. However, the particle size and distribution of the drug in nanocrystals change constantly when the drug is put to rest. The results of the aforementioned study showed that the diameter of the UA nanosuspensions increased to 199.5 nm after 49 days at 4°C. In order to keep the particles in the suspension stable and prevent agglomeration or settlement, it is necessary to add a proper stabilizer to the suspension. However, it should be mentioned that the nanocrystalline was prepared without a stabilizer, and the desired stability was obtained nonetheless. The specific principle needs to be studied further. It is worth noting that the stability may be temporary, because the polymer dispersion coefficient value also increases to a certain extent as the grain size increases to 199.5 nm (Song et al., 2014). Pi et al., used poloxamer 188 as a stabilizer and prepared short bar-shaped UA nanocrystals with an average particle size of 291.7 nm and bar-shaped UA microcrystalline with an average particle size of 1,299.3 nm using the high-pressure homogenization method. Compared with the ordinary UA suspension, the dissolution rate of nanocrystals was better and the bioavailability of nanocrystals was 2.56 times higher (that of microcrystalline was only 1.40 times higher) (Pi et al., 2016). Wang et al. (2015) evaluated the effect of UA nanocrystals with different particle sizes on the proliferative activity of MCF-7 breast cancer cells *in vitro*. The results showed that both early and late apoptotic rates were significantly higher for nanocrystals with a particle size of 300 nm than for those with a particle size of 100 nm. This may be related to the change in adhesion, which increases the contact time with cells and promotes the phagocytosis of particles by cells. However, a small particle size is generally more conducive to dissolution, so there may be a nanocrystal size with the best efficacy.

### Carrier-Free Nanoformulations Containing Indocyanine Green, a Photosensitizer

Indocyanine green (ICG) is a clinical near-infrared light (NIR) fluorescent dye with excellent biocompatibility and approved by the FDA. It has been applied in many fields, such as the imaging diagnosis of diseases and photothermal therapy (Chen T. et al., 2018; Li C. et al., 2020). Nanoparticles loaded with ICG, UA or other chemotherapy drugs can promote the efficient release of chemotherapy drugs and, at the same time, achieve accurate localization of the lesion, which has great application potential for the diagnosis of tumor diseases.

Guo et al., combined UA, PTX, and ICG into a carrier-free spherical nanoparticle (ICG @ UA/PTX NPs) with a particle size of approximately 130.8 nm and good stability, even if stored in an aqueous solution for 20 days. This may be due to the electrostatic and hydrophobic interactions between the co-assembled nanoparticles. After 5 min of NIR laser irradiation, the temperature of the NPs increased from 39 to 65°C. The cell test showed that, compared with NIR-free cells, the nanoparticles had more significant cytotoxicity on HepG2 cancer cells and HeLa cells under the action of NIR laser. The H22 tumor-bearing mouse model was used in the animal experiments. The results showed that the tumor volume of mice exposed to NIR nanoparticles decreased significantly compared with that of other groups, and there was no tumor recurrence during the 21-days observation period. Although the study showed that a large number of ICG @ UA/PTX NPs were endocytosed by HepG2 cancer cells within 3 h, in another study, the prepared nanoparticles were added with hepatotargeting LA molecules. Finally, the nanoparticles UA-LA-ICG NPs were prepared, making the intracellular fluorescence intensity stronger (Zhao et al., 2018). The death rate of HepG2 cells was 95%, and the tumor inhibition rate of the H22 tumor-bearing mouse model was 96.32%. Notably, neither nanoparticle was observed to have tumor recurrence in the mouse model and did not cause major organ damage in the mice (Guo et al., 2017).

### Self-Assembled Nanoformulations Combined With Chemotherapeutic Agents

The UA nanoformulations, combined with chemotherapeutic agents, allows for more precise release of cargo to its destination. And depending on the design, it can be released simultaneously or sequentially during transmission, creating synergies and enhancing therapeutic effects. UA was chemically conjugated to low molecular weight heparin (LMWH) to produce micellar LMWH-UA, which improves the efficacy of LMWH against tumor angiogenesis and reduces the risk of bleeding in cancer therapy. Experimental observations have demonstrated that LMWH-UA successfully reduces the risk of bleeding by reducing the affinity of antithrombin. The degradation of LMWH-UA in the plasma was 6.64 ± 0.87% after 72 h, indicating that LMWH-UA has good stability in the plasma. LMWH-UA shows high affinity to mouse melanoma B16F10 cells through specific polypeptide-mediated endocytosis (Cheng W. et al., 2017).

Furthermore, UA is a natural molecule with self-assembly properties, which can combine with other chemical molecules to form supramolecular compounds with specific structures and properties (Lu et al., 2019; Grosu et al., 2020). Paclitaxel (PTX) is one of the chemotherapeutic drugs widely used in clinic. Wang et al., prepared nanoparticles UA-PTX by means of intermolecular hydrogen bonding and hydrophobic interaction through self-assembly, which had good stability and could be rapidly absorbed by tumor cells, and basically realized complete drug loading under the coordination of intermolecular forces without showing the phenomenon of explosive release. This was confirmed by subsequent experimental observations. The half-life of UA-PTX nanoparticles was significantly longer *in vivo*, about six times that of PTX alone. In addition, UA and PTX were observed to block MCF-7 tumor cell proliferation and induce apoptosis through different mechanisms, respectively, both of which had synergistic anti-tumor effect and reached a tumor inhibition rate of 90% (Wang et al., 2020b).

## Clinical Studies of UA Nanoformulations

Clinical study is a systematic study of a drug in humans (patients or healthy volunteers) with the purpose of determining the efficacy and safety of the drug under test (De Rycker et al., 2018). Although the good anticancer effect of UA nanoformulations has been confirmed in cell or animal preclinical experiments, clinical studies on UA nanoformulations are still insufficient and need to be further explored. Xia et al. enrolled 8 healthy volunteers in a single-dose study of ursolic acid liposomes (98 mg/m^2^) in 2010, however, only a few pharmacokinetic parameters were observed to demonstrate the success of the new detection methods. The values of maximum plasma concentration (*C*_max_), time to maximum plasma concentration (*T*_max_), half-life (*t*_1/2_), area under the plasma concentration time curve (AUC_0→t_), and AUC_0→∞_, were 3,404.6 ± 748.8 ng/mL, 4.0 ± 0.0 h, 3.9 ± 2.1 h, 9,644.1 ± 1,193.2 ng h/mL, 9,918.4 ± 1,215.2 ng h/mL, respectively (Xia et al., 2011). Zhu et al. enrolled 24 healthy volunteers and 8 tumor patients in the single-dose PK study and the multiple-dose PK study, respectively. Twenty-four volunteers in the single-dose study were randomly assigned to 37, 74, and 98 mg/m^2^ doses, while eight patients in the multiple-dose study received 74mg/m2 doses for 14 consecutive days. The *C*_max_ and AUC_0→16h_ increased linearly with dose escalation. But in the multiple-dose study, The *C*_max_, *T*_max_ and t1/2 on the first day was similar to those on the 14th day (*C*_max_ 1,589 ± 635 vs. 1,211 ± 204 ng/mL, *T*_max_ 3.00 ± 1.41 vs. 3.63 ± 1.06 h, and t_1/2_ 4.58 ± 2.04 vs. 4.00 ± 1.27 h), indicating to some extent that no drug accumulation was observed after repeated administration. In the whole study, UA liposom-related adverse events were mostly mild to moderate, and the common adverse reactions were nausea, diarrhea and abdominal distension, indicating that UA liposomes were well tolerated (Zhu et al., 2013). Wang et al. enrolled 63 subjects for a single-dose study of UA liposomes, which also presented linear values of pharmacokinetic parameters. Moreover, this study showed that the maximum tolerated dose of UA liposomes was 98 mg/m^2^ (Wang et al., 2013). In 2015, Qian et al. conducted a phase Ⅰ clinical trial with 21 participants to evaluate the efficacy and tolerability of UA liposomes. The trial observed that about 60% of patients stabilized after two cycles of treatment, and indicated that UA liposomes did not accumulate *in vivo*. Furthermore, this trial concluded that the recommended dose of UA liposomes was 98 mg/m^2^, of course, which would need to be confirmed by a phase Ⅱ clinical trial (Qian et al., 2015).

## Current Situation and Future Prospects of UA Nanoformulations

### Current Situation

**FIGURE 4:**
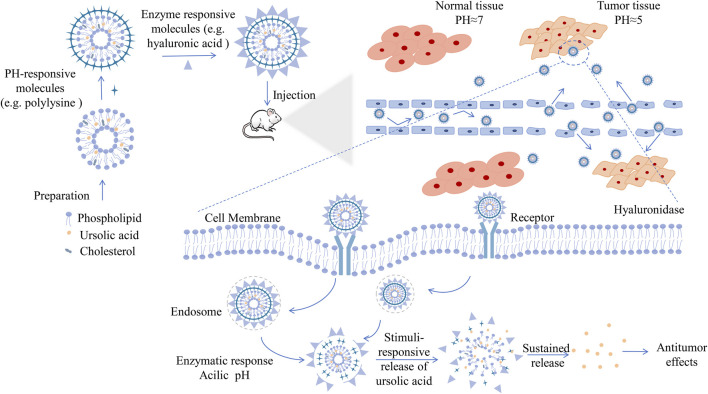
Schematic diagram of UA nanoformulations from preparation to antitumor, e.g., double-responsive liposomes. Liposomes, similar to biofilm compositions, are membrane structure composed of phospholipid bilayer and capable of chimeric cholesterol molecules, which can be prepared by classical methods, such as film hydration method, reverse-phase evaporation technique, or by other novel methods. As a hydrophobic drug, UA is located in the hydrophobic bilayer of phospholipid molecules. What's more, stepped pH and high expression of metabolic enzymes are characteristics of tumor microenvironment. By reasonably stacking different surface modification molecules, such as polylysine and hyaluronic acid, UA liposomes have the double response function of pH and enzyme, which can better cope with the special changes of microenvironment in the process of delivery, and have stronger tissue targeting to achieve better therapeutic effect. Liposomes enter and accumulate in tumor tissue through the EPR effect, the same as other nanoformulations. Liposomes enter the interior of tumor cells through cell surface receptor-mediated endocytosis. Under enzymatic reaction and appropriate acidic pH, the modified liposomes disintegrate and release the encapsulated UA molecules, playing an anti-tumor effect. Abbreviations: UA, ursolic acid.

The research of natural molecule UA in the nanofield has made great progress. Through extensive inquiry and research, we have summarized the main antitumor research achievements of current UA nanoformulations in Table 2, and have illustrated the process from preparation to anticancer effect of UA nanoformulations by taking double-responsive liposomes as an example in Figure 4. The improvement of the antitumor ability of the UA nanoformulations is mainly due to the improvement of the bioavailability and the enhancement of the targeting ability of the UA molecules. First of all, bioavailability is the rate and extent to which a drug is absorbed into the circulation of the body, which is affected by the chemical structure, solubility, hydrophilic lipophilicity, stability, dosage form characteristics and other factors of the drug molecule (Tjandrawinata et al., 2018). The stability, surface modification and the development of preparation technology of nanoformulations have greatly improved the bioactivity and bioavailability of UA molecules (Yang et al., 2012; Antonio et al., 2021). Studies have confirmed that the area under the plasma drug concentration-time curve (AUC) and the peak concentration (*C*_max_) that can be achieved after administration of UA nanoformulations *in vivo* are 2–3 times that of the original UA molecules (Pi et al., 2016; Qiu et al., 2019). Moreover, EPR effect is the basic principle of passive targeting of nanoformulations to tumor tissues. However, due to the complex internal environmental changes in organisms, it is not sufficient to rely solely on EPR effect, so molecular modification is usually carried out on the surface of nanoparticles, such as FA and lactose acid. Modified molecules act as ligands to enhance the targeting activity of nanoformulations and endocytosis of tumor cells by interacting with tumor cell surface receptors. With the help of fluorescence imaging technology, it has been proved that modified UA nanoparticles are more easily distributed in tumor tissues and display strong fluorescence images (Jin et al., 2016a; Zhao et al., 2018). Finally, UA combined with other chemotherapy drugs or other natural anticancer molecules has synergistic therapeutic effects, which may be a good method for the treatment of drug resistance of cancer, because experiments have proved that combined therapy can induce apoptosis of cancer cells through different signaling pathways and reduce the use of chemotherapeutic drugs (Alvarado et al., 2018; Silva et al., 2019; Sun S. et al., 2020).

Currently, the design of UA nanoformulations is very diverse according to the purpose, whether to increase the stability of drug circulation, or to release drugs slowly and effectively, reduce the accumulation of non-targeted sites. On a certain extent, they all aimed to increase the efficacy of UA and promotes its better clinical application. Liposomes are basically perfect systems with components similar to biofilm compositions and are designed to move drug molecules across specific cell membranes, offering excellent drug delivery potential (Lee, 2020). However, due to the fluidity similar to that of biofilms, liposomes are easily affected by temperature changes *in vivo*, resulting in membrane permeability changes and drug leakage. Micelle has a unique cored-shell structure, but compared with other common types of nanomaterials, the drug loading of micelle is usually less, which may make it difficult to exert the expected therapeutic effect of UA (Siboro et al., 2020). Nanocrystals is pure drug nano preparation with a very perfect drug loading capacity, and is easy to scale up production. Moreover, nanocrystals have a wide range of applications, including transdermal, ocular, nasal, and lung drug delivery. The same is true of nanoemulsion. Nevertheless, nanoemulsion system is relatively fragile, and may be out of balance even with small changes in the environment (Jarvis et al., 2019; Naseema et al., 2021).

Also, PCL, PLA, PLGA, MSNs, chitosan, and other materials are commonly used as nano-delivery carriers for UA nanoformulations. Safety is an important driving force for the use of nanocarriers as pharmaceutical excipients. After the drug has been delivered to the site, the carrier material itself should be completely excreted within a reasonable period of time, or its metabolites should be clear and non-toxic. Chitosan is a natural cationic polysaccharide, which can make drugs enter cells by interacting with negatively charged residues in membrane proteins (Torres-Rosas et al., 2020). PLA is a kind of renewable biodegradable material derived from starch production, which eventually generates carbon dioxide and water and realizes the circulation in nature. PLA is an ideal green polymer material. PCL, which is the same as PLA metabolites, is also favored due to its excellent biocompatibility and biodegradability. It has good flexibility and processability, and is easy to form film, although its mechanical strength is slightly insufficient (Zhai et al., 2017; Brčić et al., 2021). PLGA is formed by the polymerization of two kinds of monomers including PLA. Its performance is related to the proportion of monomers. The lower the proportion of PLA, the easier the degradation of PLGA. PLGA has good biocompatibility and has been certified as a safe medical excipient (Houshmand et al., 2020). Different from PCL, PLA, PLGA and other polymer materials, MSNs is an inorganic material, which is hard to be degraded by biological enzymes in the body, so it can reduce the drug loss in the delivery process. And it is convenient for surface functional modification. Significantly, the stability of the nanoparticle circulation *in vivo* was enhanced after the encapsulation of the carrier. However, the carrier occupies a large proportion in the whole nanosystem, which affects the drug loading efficiency of nanoformulations (Wang et al., 2020a). Therefore, the nanosystem assembled without carrier emerges as the times require, and it can combine with photosensitizer to play an excellent image diagnosis and optical treatment effect.

### Future Prospects

Although research on UA nanoformulations has progressed adequately, there are still some problems to be solved. The ideal nanoformulations should be able to penetrate the core of a tumor tissue at a high drug concentration, enter the tumor cells where the target molecules are located, completely eradicate the tumor, and improve the survival rate of patients. This involves five steps: entering the blood circulation, enriching the tumor site, infiltrating the tumor tissue, reaching the tumor cells, and being ingested by them. Finally, the drug is released inside the cell (Dong et al., 2019). Tumor tissue penetration is an indispensable and important link for the efficacy of nanoformulations (Wadajkar et al., 2019).

The permeability of nanoformulations is related to the heterogeneity of the tumor microenvironment (Feng and Mumper, 2013; Vong and Nagasaki, 2020). Nanoformulations’ diffusion is also limited by the pressure of the interstitial fluid in the tumor microenvironment after passing through the tumor neovascularization wall from the basement membrane defect. Although nanoformulations can respond to changes in pH or enzymes in the microenvironment with a single or multifunctional modification, the permeability can be improved to a certain extent; however, studies have shown that this improvement is inefficient. This may be because the large size of the nanoformulations makes it difficult to move around in complex microenvironments compared to small molecules. Nanopreparation modifications have not progressed greatly. Therefore, is it feasible to reduce the pressure of the tumor microenvironment intermediate fluid? The answer is no. Owing to changes in the interstitial fluid in the microenvironment, cancer cells may be more susceptible to invasion and metastasis, which to some extent defeats the purpose of cancer treatment. A significant step forward and a potential answer to this dilemma, may be the findings of certain studies that have shown that adenosine triphosphate, which plays an important role in various physiological activities of cells, can act as a stimulant to control drug release in some tumor tissues. Thus, to some extent, the permeability of nanoformulations in tumor tissue active transport may be improved by the way of active transport based on the energy consumption of adenosine triphosphate (Moradi Kashkooli et al., 2020; Zhou et al., 2020).

As research progresses, the delivery efficiency of UA nanoformulations may be improved significantly; however, there is still a long way to go before clinical application. To date, the physicochemical properties and metabolic behavior of UA nanopreparations *in vivo* have not been fully elucidated, so in-depth preclinical experimental and clinical trial explorations are necessary. Establishing large animal models of various cancer types, or combining mathematical models to simulate and track the *in vivo* behavior of UA nanoformulations may be beneficial to further evaluate their *in vivo* characteristics (He et al., 2020). Another important indicator of the clinical application of UA nanoformulations is the quantification of production (Feng et al., 2019). At present, promoting the efficient transportation of nanoformulations is achieved mainly by gathering different functional groups into the same polymer, which increases the complexity of nanoformulations' structure and further reduces the controllability of their large-scale production. Ideally, a modified nanoparticle with one group would complete the entire delivery process, which would be a good clinical transformation.

## Conclusion

**FIGURE 5:**
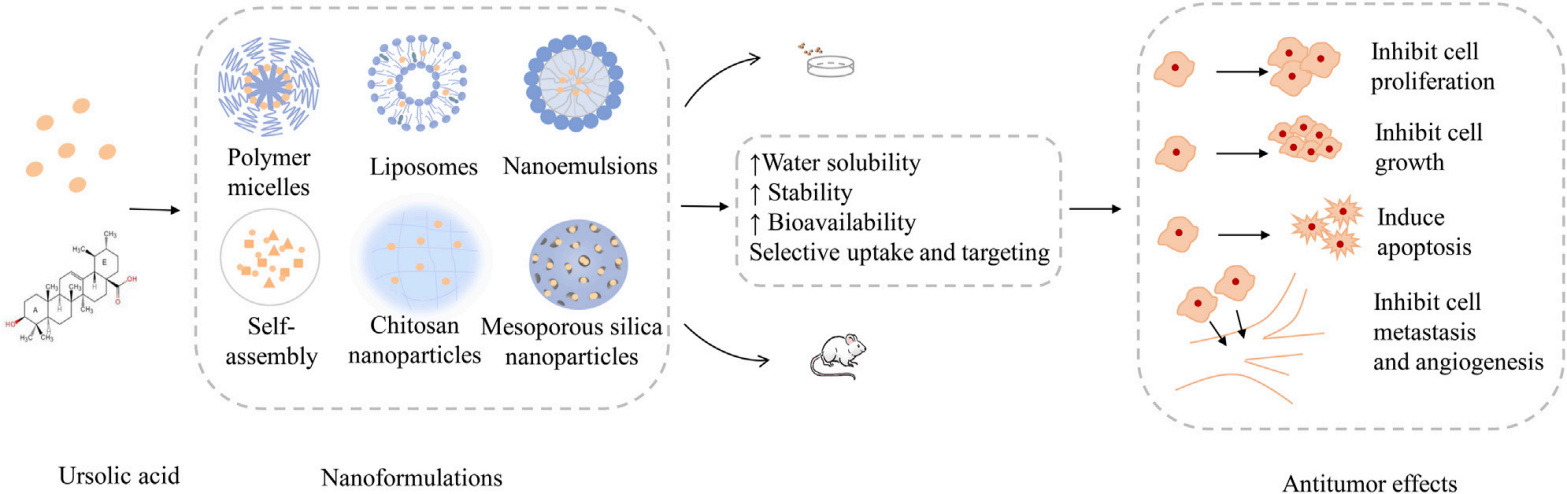
The main properties of UA nanoformulations and their therapeutic efficacy. UA nanoformulations are designed in a variety of ways, such as, polymer micelles, liposomes, nanoemulsions, nanoparticles, etc. Confirmed by a large number of cell and animal experiments, the UA nanoformulations can improve the water solubility, stability, bioavailability and tissue targeting of UA molecules, and achieve good anti-tumor effect by inhibiting the growth, proliferation and metastasis of tumor cells, promoting tumor cell apoptosis and inhibiting tumor angiogenesis. Abbreviations: UA, ursolic acid.

This review highlighted the advantages and disadvantages of UA as a natural plant-based anticancer compound, indicating that new cancer treatment strategies based on nanotechnology for UA have broad clinical application prospects. The mature application of nanotechnology in the field of medicine has smoothed the path of cancer treatment. Based on the EPR effect, nanoformulations selectively concentrate in tumor tissues and reduce the distribution of normal tissues, which, to some extent, enhances the anticancer efficacy of drugs and reduces drug-induced adverse reactions. Currently, a variety of UA nanoformulations, such as micelles, liposomes, nanoparticles, and nanocrystals, which have higher stability, better absorption rates, and higher cancer cell lethality than UA compounds, have been prepared successully. The main properties of UA nanoformulations and their therapeutic efficacy are shown in Figure 5. Despite the current research on the nanoformulations is still in the laboratory stage, with the continuous thinking and hard exploration of the vast number of researchers and engineers, a stable, efficient, safe and industrially reproducible ideal UA nanoformulations is worth looking forward to.
